# Social Determinants of Health and Health Literacy in Emergency Patients with Diabetic Ketoacidosis

**DOI:** 10.5811/westjem.35262

**Published:** 2025-03-31

**Authors:** Daniel F. Suarez, Ryan M. Schneider, Margo Girardi, Gina LaRossa, Julianne Yeary, Taylor Kaser, Rachel Ancona, Paulina Cruz Bravo, Richard T. Griffey

**Affiliations:** * Washington University School of Medicine, Barnes-Jewish Hospital, Department of Emergency Medicine, St. Louis, Missouri; † Washington University School of Medicine, Barnes-Jewish Hospital, Department of Internal Medicine Divisions of Hospital Medicine, St. Louis, Missouri; ‡ Washington University School of Medicine, Barnes-Jewish Hospital, Department of Endocrinology, St. Louis, Missouri

## Abstract

**Introduction:**

Social determinants of health (SDoH) and health literacy have been demonstrated to significantly impact health outcomes. As part of a study of diabetic ketoacidosis (DKA) treatment from the emergency department (ED), we assessed the burden of SDoH and health literacy among patients with DKA to identify potentially modifiable risk factors in the development of DKA.

**Methods:**

This was an exploratory, prospective, cross-sectional study of adult patients with DKA in a large urban academic ED from March 2023–March 2024. We administered the Centers for Medicare & Medicaid Services Accountable Health Communities Health-Related Social Needs Screening Tool (SNST) and the Brief Health Literacy Screen (BHLS).

**Results:**

Of 126 identified ED patients with confirmed DKA, 57 completed the SNST and 72 completed the BHLS. Nearly all patients (56 patients, 98%) reported at least one unmet SDoH need, and 32 (56%) patients reported five or more. The most frequently reported SDoH needs were physical activity (77%), mental health (63%), financial strain (60%), substance use (54%), and food insecurity (51%). Seventy-two patients completed the BHLS, which demonstrated high levels of health literacy, with median responses ranging from 4–5 on a Likert scale with 5 corresponding to highest health literacy.

**Conclusion:**

Social determinants of health needs are prominent among patients who develop DKA, highlighting an opportunity for ED-based interventions to address specific SDoH factors to prevent the development of this disease. Self-reported health literacy scores were high in this patient population.

## INTRODUCTION

Diabetes is one of the most significant diseases afflicting the global population, affecting 8.5% of adults aged 18 years or older.^1^ Diabetic ketoacidosis (DKA) is the most common acute hyperglycemic emergency in patients with diabetes and is associated with over 200,000 annual emergency department (ED) visits in the United States,^2^ with a mortality rate of 2–5%.^3^ Hospitalization for DKA is costly and traumatic for individuals; it is on the rise but often preventable. The development of DKA is related to poor glycemic control and has been found to disproportionately affect racial and social minorities.^1^,^4^ The contemporary study of diabetes and DKA is expanding focus to emphasize human factors and population-based outcomes to better understand the socioeconomic distribution of patients who present in DKA. Increasingly, prevention efforts are focusing on social determinants of health (SDoH) that contribute to acute exacerbations of chronic disease.

The World Health Organization defines SDoH as “non-medical factors that influence health outcomes” including “the conditions in which people are born, grow, work, live and age, and the wider set of forces and systems shaping the conditions of daily life.”^5^ Poor SDoH are associated with negative diabetes-related outcomes including the development of DKA.^6^ Personal health literacy—the degree to which individuals understand and use information and services to inform health-related decisions^7^—is an important interface between the healthcare system and the public. Low scores on health literacy measures correlate with poor health outcomes including increased ED utilization and hospital admission.^8^–^10^ Varying levels of health literacy can limit patients’ understanding of their disease process and limit participation in their healthcare plan, which illustrates a modifiable risk factor for the improvement of their health.

At our institution, we implemented a new treatment protocol for mild-to-moderate DKA that uses subcutaneously administrated insulin instead of the traditional insulin infusion, which has been successful in safely treating patients with DKA in a non-intensive care unit setting.^11^ Implementation of this new treatment initiative provided an opportunity for us to examine health literacy and SDoH data with the aim to identify potentially modifiable risk factors leading to the development of DKA in our patient population.

## METHODS

### Design, Setting, Participants

As part of a broader study related to treatment of DKA, we collected data on SDoH burdens and self-reported health literacy in patients with DKA. This prospective, cross-sectional study took place over one year (March 2023–March 2024) in a large, urban, academic ED. Electronic surveillance notified a research assistant (ML) to contact the patient by telephone while in the hospital for consent and data collection. This study was approved by our hospital institutional review board.

### Measurements

We used the Centers for Medicare & Medicaid Services Accountable Health Communities Health-Related Social Needs Screening Tool (SNST), where patients answer a 26-item questionnaire spanning 13 domains of SDoH.^12^ We dichotomized patient responses for each domain into “one or more unmet needs” or “no unmet needs” according to the tool guidelines. We also employed the Brief Health Literacy Screen (BHLS), a three-item questionnaire that evaluates self-reported health literacy using a five-point Likert scale from 1 (lowest health literacy) to 5 (highest health literacy).^13^

**Population Health Research Capsule**

What do we already know about this issue?

*The development of diabetic ketoacidosis (DKA) has been found to disproportionately affect racial and social minorities.*

What was the research question?

*Can we identify specific social determinants of health (SDoH) as potentially modifiable risk factors in the development of DKA?*

What was the major inding of the study?

*98% of patients (56/57 patients, 95% CI 89*–*100%) reported at least one unmet SDoH need, and 56% (32 patients) had five or more.*

How does this improve population health?

*We identified several SDoH burdens among ED patients presenting in DKA, which represent potentially modifiable risk factors for the development of DKA.*

### Data Analysis

Because this was an exploratory analysis we did not perform a sample size calculation but aimed to enroll patients over a one-year period. In an attempt to determine whether patients completing each questionnaire were similar in demographics to DKA patients who did not complete the surveys (patients who could not be reached for survey participation and patients who initiated the surveys but did not complete them), we used the Mann-Whitney U test to compare age and chi-squared tests and the Fisher exact test to compare sex, race, and ED disposition, where appropriate. We present summary descriptive statistics, reporting frequencies and proportions for the prevalence of all SDoH categories among our patient population and medians and interquartile ranges (IQR) for BHLS scores. We conducted all data management and computed descriptive statistics in R version 4.3.1, employing the packages tidyverse versions 1.3.0 and 1.4.3 (R Foundation for Statistical Computing, Vienna, Austria).^14^,^15^

## RESULTS

**Figure 1:**
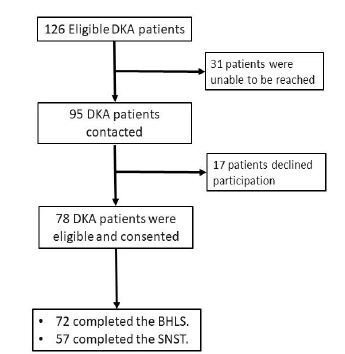
Study flow diagram. *BHLS*, Brief Health Literacy Screen; *DKA*, diabetic ketoacidosis; *SNST*, Social Needs Screening Tool.

**Figure 2:**
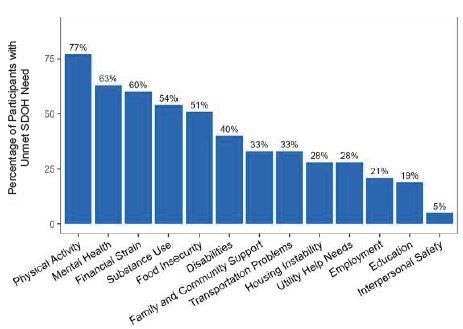
Unmet social determinants of health needs by category in patients with diabetic ketoacidosis. *SDOH*, social determinants of health.

Among 95 patients with DKA contacted, 78 consented to participate with 57 (60% response rate) completing the SNST and 72 (76% response rate) completing the BHLS (Figure 1). Participants completing the SNST were similar to the 36 eligible non-respondents with DKA (31 patients who could not be reached and fivc who initiated the surveys but did not complete them) in terms of age, sex, race, and ED disposition (all *P* > 0.96) (Table 1). Of the 57 patients, 56 (98%, 95% confidence interval [CI] 89–100%) reported at least one unmet SDoH need, and 32 (56%) patients had five or more. Of the identified unmet SDoH needs, the most frequently reported were physical activity (77%), mental health (63%), financial strain (60%), substance use (54%), and food insecurity (51%) (Figure 2).

Patients who completed the BHLS were similar to non-respondents in terms of age, sex, race, and ED disposition (all *P* > 0.90). For patients completing the BHLS, health literacy was high, with median responses to all three questions ranging from 4–5 on a Likert scale with 5 corresponding to highest health literacy (Table 2).

## DISCUSSION

Social determinants of health are increasingly recognized as significant contributors in the development of DKA. Identifying potentially modifiable factors may be the first step in helping prevent the development of DKA altogether. Using the SNST inventory, we identified several prominent and potentially modifiable SDoH burdens across several domains among ED patients presenting in DKA. Greater than 50% of patients identified physical activity, mental health, financial strain, food insecurity, and substance use as areas of need. Physical activity was the most prominent area of identified SDoH need, affecting over three-quarters of our study population. These results are consistent with previous studies, which have identified specific SDoH factors correlating to higher incidence of DKA including area-level economic deprivation^16^ and substance use.^17^ Hamblin et al similarly found recurrent episodes of DKA to be associated with unemployment, low education level, less medical contact, and drug and tobacco use.^18^

Diabetic ketoacidosis in patients with type 1 diabetes mellitus has also been associated with increased rates of suicide, particularly within 12 months of the DKA episode.^19^ We were surprised to find health literacy scores were high, despite the high rates of self-reported SDoH burdens. In prior evaluations of health literacy in our ED we found approximately 24% of ED patients had limited or poor health literacy.^20^ Objective health literacy assessment tools are generally preferred over self-reported instruments, which are felt to be more accurate, but the brief health literacy questions fared well in the ED in our prior study.^21^

In their 2021 review of SDoH and diabetes, Hill-Briggs noted a paucity of US-based research examining the impact of interventions designed to target education, income, occupation, toxic environmental exposures, social cohesion, and social capital on diabetes outcomes.^6^ Since this publication, a number of studies have evaluated the impact of interventions targeted at various SDoH factors on outcomes in diabetes. For example, “produce prescription programs,” which provide vouchers for fresh fruits and vegetables and diabetes education are one area being investigated for their impact on diabetes outcomes including reductions in hemoglobin A1C levels, although results are mixed.^22^,^23^ Educational campaigns to increase awareness of the importance of physical activity and encourage home exercise and lifestyle modifications to prevent the development of type 2 diabetes have also been described.^24^ However, we are not aware of studies that have specifically explored the impact of interventions on the development of DKA.

Discussions in the diabetes literature also focus on policy, research, and practice pattern adjustments that may reduce diabetes-related health disparities, including the development of DKA. A 2024 international consensus report recognizes SDoH as a “major risk factor” for the development of DKA or hyperosmolar hyperglycemic state and recommends screening for SDoH prior to discharge in patients admitted for DKA. They further assert: “In the USA, policy solutions such as increasing access to health insurance, affordable insulin, medical care, nutritious food and housing would be expected to reduce the incidence of DKA.”^25^ Levi et al explore specific paths toward improving health outcomes through federal and community-based initiatives aimed at improving glycemic control in patient with diabetes. They propose strategies to expand “food-is-medicine” programs (produce prescriptions, medical meal-delivery platforms), strengthen existing federal nutrition assistance programs such as the Supplemental Nutrition Assistance Program and Special Supplemental Nutrition Program for Women, Infants and Children, and emphasize the importance of future research to understand the ideal dose and duration of nutritional support programs.^26^

## LIMITATIONS

This was a single-center study with potential limitations related to generalizability. As this study was exploratory, a sample size calculation was not performed. We aimed to enroll 100 patients, but this number was somewhat arbitrary. Our sample size was lower than anticipated, in part due to a lower volume of patients with DKA during the study period compared to historical averages. In addition, although prior research experience favored the use of telephone contact of patients while in the hospital, we faced some limitations in this area, impacting our enrollment. An evaluation at one year of data collection noted marked stability and lack of variation in our results over time with a low likelihood that additional data would have impacted our results. This prompted us to truncate data collection at that point, which coincided with the end of our funding period.

Additionally, the self-reported nature of these data is a limitation given its reliance on personal perception and vulnerability to recall, social desirability bias, selection bias, and other biases. This work was also limited by the lack of a control group to determine which identified SDoH burdens are unique to ED patients with DKA vs being reflective of our population more broadly. We did attempt but discontinued an effort to capture SDoH information among type 1 diabetes patients not in DKA in the ED due to exceedingly low numbers.

A study comparing SDoH and health literacy among diabetic patients who develop DKA vs those who do not would provide interesting context to the impact of these social factors on glycemic control. A 2011 study found that poor adherence to insulin therapy impacted by behavioral, socioeconomic, psychosocial, and educational factors was the leading cause of recurrent admissions for DKA in urban patients from racial and ethnic minorities.^27^ A survey of DKA patients on perceptions of the factors that led to the development of DKA and factors impacting their glycemic control, including missed insulin doses, concomitant illness, and/or polypharmacy would be useful. We expect there may be opportunities for local initiatives to make meaningful impacts on selected SDoH burdens.

Our future work will focus on further investigation, perhaps including qualitative methods and obtaining more detailed information about needs within the broad SDoH domains identified and the degree to which these are modifiable. We also anticipate partnership with our social work, addiction, and mental health colleagues to identify screening, brief intervention, and referral for treatment approaches to improving substance use and mental health burdens identified.

## CONCLUSION

We identified several potentially modifiable burdens of social determinants of health among emergency patients with diabetic ketoacidosis. Further study is needed to identify more specific needs, develop interventions to address these burdens, and determine whether these interventions may reduce recurrence of DKA.

## Figures and Tables

**Table 1:** Sociodemographics of patients with diabetic ketoacidosis.

	DKA patients who did not participate (N=36)	DKA patients who completed the BHLS (N=72)	DKA patients who completed the SNST (N=57)
Age (years) – median (IQR)	43 (28, 64)	47 (33, 57)	48 (32, 57)
Sex – n (%)			
Male	22 (61)	42 (58)	33 (58)
Female	14 (39)	30 (42)	24 (42)
Race – n (%)			
Black	24 (67)	52 (72)	40 (70)
White	11 (31)	15 (21)	13 (23)
Other	1 (3)	1 (1)	1 (2)
Not available	0 (0)	4 (6)	3 (5)
ED disposition – n (%)			
Discharged from the ED	1 (3)	6 (8)	4 (7)
Admitted to observation floor	14 (39)	31 (43)	24 (42)
Admitted to medical floor	8 (22)	11 (15)	9 (16)
Admitted to ICU	13 (36)	24 (33)	20 (35)

The group of individuals who did not participate is comprised of 31 patients who could not be reached for survey participation and five who initiated but did not complete either survey and, thus, were dropped from the analysis.

*BHLS*, Brief Health Literacy Screen; *ED*, emergency department; *ICU*, intensive care unit; *IQR*, interquartile range; *SNST*, Social Needs Screening Tool.

**Table 2:** Health literacy survey results of patients with diabetic ketoacidosis.

BHLS screening question	Median (IQR) (N=72)
How often do you have problems learning about your medical condition because of difficulty understanding written information?	4.00 (3.00, 5.00)
How confident are you filling out medical forms by yourself?	5.00 (4.00, 5.00)
How often do you have someone help you read hospital materials?	4.00 (3.00, 5.00)
